# Analysis of the global transcriptome of longan (*Dimocarpus longan* Lour.) embryogenic callus using Illumina paired-end sequencing

**DOI:** 10.1186/1471-2164-14-561

**Published:** 2013-08-19

**Authors:** Zhongxiong Lai, Yuling Lin

**Affiliations:** 1 Institute of Horticultural Biotechnology, Fujian Agriculture and Forestry University, Fuzhou, Fujian 350002, China

## Abstract

**Background:**

Longan is a tropical/subtropical fruit tree of great economic importance in Southeast Asia. Progress in understanding molecular mechanisms of longan embryogenesis, which is the primary influence on fruit quality and yield, is slowed by lack of transcriptomic and genomic information. Illumina second generation sequencing, which is suitable for generating enormous numbers of transcript sequences that can be used for functional genomic analysis of longan.

**Results:**

In this study, a longan embryogenic callus (EC) cDNA library was sequenced using an Illumina HiSeq 2000 system. A total of 64,876,258 clean reads comprising 5.84 Gb of nucleotides were assembled into 68,925 unigenes of 448-bp mean length, with unigenes ≥1000 bp accounting for 8.26% of the total. Using BLASTx, 40,634 unigenes were found to have significant similarity with accessions in Nr and Swiss- Prot databases. Of these, 38,845 unigenes were assigned to 43 GO sub-categories and 17,118 unigenes were classified into 25 COG sub-groups. In addition, 17,306 unigenes mapped to 199 KEGG pathways, with the categories of Metabolic pathways, Plant-pathogen interaction, Biosynthesis of secondary metabolites, and Genetic information processing being well represented. Analyses of unigenes ≥1000 bp revealed 328 embryogenesis-related unigenes as well as numerous unigenes expressed in EC associated with functions of reproductive growth, such as flowering, gametophytogenesis, and fertility, and vegetative growth, such as root and shoot growth. Furthermore, 23 unigenes related to embryogenesis and reproductive and vegetative growth were validated by quantitative real time PCR (qPCR) in samples from different stages of longan somatic embryogenesis (SE); their differentially expressions in the various embryogenic cultures indicated their possible roles in longan SE.

**Conclusions:**

The quantity and variety of expressed EC genes identified in this study is sufficient to serve as a global transcriptome dataset for longan EC and to provide more molecular resources for longan functional genomics.

## Background

Longan (*Dimocarpus longan* Lour.), a tropical/subtropical fruit tree in the family Sapindaceae, is of great economic importance in Southeast Asia. Because the status of embryo development determines seed size, fruit quality, percentage of fruit set, and yield in longan, efforts to improve fruit quality and yield have included studies on regulation of longan embryo development using cytological, molecular, and proteomics approaches [1,2]. Such research has been hampered, however, by the extremely high genetic heterozygosity of longan and early embryo sampling difficulties [3]. Because plant somatic embryogenesis (SE) shows close similarities on morphological and molecular levels to normal zygotic embryogeny [4-7], the longan SE system has been used as a system for investigating regulation of *in vitro* and *in vivo* embryogenesis in longan [8-10]. Studies focusing on molecular biology and proteomics of the longan SE system have been conducted using differential display reverse transcription PCR (DDRT-PCR), homology cloning, quantitative real-time PCR (qPCR), two-dimensional electrophoresis, and protein bio-mass spectrometry (MALDI-TOF, Q-TOF), resulting in the isolation and identification of hundreds of related genes and proteins [1].

But little genomic or proteomic information of the above-mentioned studies is available for the longan embryo. As of July 2013, only 652 nucleotide sequences and 66 expressed sequence tags (ESTs) had been deposited in the NCBI GenBank database. Although many key longan genes and proteins have been cloned and identified, molecular resouces of longan are still limited because genomic and transcriptomic information is lacking. Consequently, an accelerated effort to acquire transcriptomes of longan embryogenesis is needed. A few transcriptomic studies of embryogenesis have been conducted in rice [11,12], poplar [13,14], *Arabidopsis*[15,16], *Gossypium hirsutum*[17], *Solanum tuberosum*[18], *Elaeis guineensis*[19], *Brassica napus*[20]*,* soybean [21], and maize [22]; these studies were mainly focused on calli or embryogenic calli, and involved techniques such as Illumina sequencing, massively parallel signature sequencing (MPSS), EST analysis, microarray analysis, and suppression subtraction hybridization (SSH). No research has been performed on the longan transcriptome.

To assist in the identification, quantification, and classification of genes expressed in longan embryogenic callus (EC), we generated a global transcriptome from longan EC using high-throughput Illumina RNA sequencing, and analyzed functions, classification, and metabolic pathways of the resulting unigenes using bioinformatics. We then comparatively analyzed expression patterns to reveal 23 selected unigenes participating in longan SE. The resulting assembled and annotated transcriptome should serve as a highly useful resource for the identification of genes involved in longan SE.

## Results

### Illumina sequencing, *de novo* assembly, and sequence analysis of the *D. longan* transcriptome

To obtain a global overview of the longan EC transcriptome, we constructed a cDNA library from a longan EC RNA sample. Using an Illumina HiSeq 2000 sequencing system, 64,876,258 clean reads (comprising 5.84 Gb of nucleotide data) were obtained after removing low-quality reads and adaptor sequences. Q20, N, and GC percentages were 95.88%, 0.01%, and 45.54%, respectively (Table 1).

**Table 1:** Summary of sequence assembly after Illumina sequencing

	**Sequences (n)**	**Base pairs (bp)**	**Mean length (bp)**	**N50 (bp)**
Clean reads	64,876,258	5,838,863,220	‐‐‐	‐‐‐
Contigs (≥75 bp)	491,067	67,999,370	138	98
Scaffold sequences (≥100 bp)	96,251	34,216,073	355	495
Total unigenes (≥100 bp)	68,925	30,887,508	448	572

Using the SOAPdenovo assembly program, all high-quality reads were assembled into 491,067 contigs longer than 75 bp, with a median length of 138 bp and an N50 of 98 bp. The size distribution of these contigs is shown in Additional file 1. The length of 380,516 contigs (77.49%) ranged from 75 to 100 bp; 15,556 contigs (3.17%) were longer than 500 bp, and the remaining were mainly between 200–499 bp in length.

Using a paired-end sequencing strategy, contigs from the same transcript can be identified and the distances between these contigs evaluated. 96,251 scaffolds, with a median length of 355 bp and an N50 of 495 bp, were generated (Table 1). Length distributions of the resulting scaffolds were as follows: 100–500 bp (79,339; 82.42%), 500–1000 bp (11,221; 11.66%), 1000–2000 bp (4,561; 4.47%), and >2000 bp (1.17%) (Additional file 1). Then, the ratio of gap length to length of scaffold was analyzed; 81,058 (84.22%) scaffolds had no gap at all, 10,216 (10.61%) had gap lengths less than 10% and only 1,018 (1.06%) exhibited gap lengths ranging from 20–40% of the total length.

Finally, paired-end reads were used again for gap filling of scaffolds to generate unigenes with the smallest number of Ns. 68,925 unigenes, with an average length of 448 bp and an N50 of 572 bp, were constructed from the scaffolds (Table 1). Unigenes with lengths ranging from 100–500 bp, 500–1000 bp, and 1000–2000 bp accounted for 75.44% (51,999), 16.29% (11,230), and 6.63% (4,567) of the total, respectively; in addition, 1,129 (1.64%) unigenes were ≥ 2000 bp long (Additional file 1). Of the 68,925 unigenes, 90.67% (62,492) had no gap and 5.99% (4,128) had gap lengths less than 10% of the total length.

### Protein coding region (CDS) prediction of the *D. longan* transcriptome

To determine the function of longan embryogenic unigenes, BLASTx alignment (*E*-value ≤ 1 × 10^-5^) between unigenes and Nr, Swiss-Prot, KEGG and COG protein databases was carried out, and the results were used to predict unigene transcriptional orientations and coding regions. A total of 41,644 unigenes (20,999 in sense and 20,645 in antisense orientations) were identified in the longan EC library, with 27,281 unigenes remaining unidentified.

For validation and annotation of gene names, CDS, and predicted proteins, all assembled unigenes were first searched against Nr and Swiss-Prot databases using BLASTx. In total, 55.94% (38,555) of the putative protein unigenes showed significant similarity to known plant proteins in the databases. The distribution of unigenes with homologous matches was 200–500 bp (27,881; 72.31%), 600–1000 bp (7,101; 18.42%), 1100–3000 bp (3,466; 8.99%), and >3000 bp (107; 0.28%). Furthermore, 37,719 (97.83%) unigene CDSs had no gaps at all and 610 (1.79%) exhibited gap lengths less than 10% of the total length. The coding region unigenes were translated into amino sequences using a standard codon table. There were 30,206 (78.35%) unigenes coding for polypeptides approximately 200 aa long and 7,567 (19.83%) with polypeptide lengths ranging from 300 to 600 aa. In addition, there were a few unigenes with polypeptide lengths greater than 1500 aa; these included unigenes coding for zinc finger family protein (Unigene14860; 2448 aa), vacuolar protein sorting-associated protein 13C (Unigene6919; 2269 aa), auxin transport protein (Unigene14063; 2052 aa), WD40 G-beta repeats (Unigene911; 1863 aa), phosphatidylinositol-4-phosphate 5-kinase family protein (Unigene9735; 1845 aa), and CAAX amino terminal protease family protein (Unigene9935; 1715 aa).

The remaining 30,370 unigenes with no homologs in the above databases were scanned again using ESTScan. 2,079 putative protein unigenes were identified, 1,897 (91.25%) with no gaps. Putative protein unigenes with lengths ranging from 200–300 bp accounted for 83.41% (1,734) of these; other approximate lengths represented were 400 bp (205), 500 bp (64), and 600 bp. Of the putative protein unigenes identified using ESTScan, 98.03% (2,038) translated to polypeptide sequences about 200 aa long. In total, 58.95% (40,634) of putative protein coding unigenes were annotated by homology analysis using Nr and Swiss- Prot databases or ESTScan predictions.

With respect to plant growth and developmental functions, analysis of unigenes longer than 1000 bp (most including the entire ORF) showed that at least 328 unigenes of embryogenesis-related genes were expressed in longan EC. Among them, pentatricopeptide repeat-containing protein genes (253 unigenes) were the most dominant group, followed by *EMB* (*Embryo defective*) family genes (42 unigenes), and then *MEE* (*Maternal effect embryo arrest*) family genes (7 unigenes). Surprisingly, in addition to the embryogenesis-related genes mentioned above, many reproductive growth-related genes were also expressed in EC, including genes related to flowering, meiosis, floral organ development, female and male gametophyte development, embryo sac development, ovule development, endosperm development, pollen tube growth, inflorescence meristem growth, floral organ number control, petal loss, and tapetum formation. Furthermore, some vegetative growth-related genes, such as those related to apical meristem growth, root growth, and mycorrhizal formation, were also expressed in EC (Table 2).

**Table 2:** Selected unigenes (≧1000 bp) related to reproductive and vegetative growth from longan EC transcriptome annotated by Nr

**Related plant organ, tissue or bioprocess (total No. of unigenes)**	**Related genes**	**No. of unigenes (≧1000 bp )**
Embryo (328 unigenes)	*PPR*; *Pentatricopeptide repeat-containing protein*, related to embryo development	253
	*EMB* (*EMBRYO DEFECTIVE*) 30, 976, 1011, 1030, 1135, 1270, 1273, 1374, 1417, 1674, 1691, 1703, 1789, 2016, 2247, 2261, 2410, 2411, 2421, 2453, 2454, 2458, 2730, 2733, 2745, 2746, 2750, 2754, 2756, 2761, 2765, 2766, 2771, 2773, 2776	42
	*MEE*; *Maternal effect embryo arrest* 47, 55, 12, 62, 40, 22	7
	*ISE* ( *Increased size exclusion limit*)1a, 2;	3
	*ISE*2(*EMB*25), related to lethal embryo, Essential protein required during embryogenesis.	
	*EYE; Embryo yellow*, is required for appropriate cell expansion and meristem organization in *Arabidopsis thaliana*. an embryo yellow (eye) mutation in *Arabidopsis* that leads to the abnormal coloration and morphology of embryos	1
	*EDD*1; *Embryo defective development* 1	1
	*LEA related proteins*	4
	*Embryogenesis transmembrane protein-like*	1
	*SERK*; *somatic embryogenesis receptor kinase*	4
	*DIE2/ALG10* family, related to embryonic	1
	*AHG*2 (*ABA-HYPERSENSITIVE GERMINATION* 2)	1
	*seed imbibition protein* 1	1
	*DNA binding / protein dimerization*, controls expression of genes during embryonic morphogenesis.	1
	*lectin-like receptor kinase* 7, regulates ABA response during seed germination	1
	*NIMA-related protein kinase*, suppresses ectopic outgrowth of epidermal cells through its kinase activity and the association with microtubules (epidermal cells of the hypocotyls and petioles)	1
	*BIO*1 (*biotin auxotroph* 1), related to embryo development	1
	*RST*1 (*Resurrection*1), related to wax metabolism and embryo development	1
	similar to *wax synthas*e/*wax synthase-like protein,* related to wax metabolism and embryo development	2
	*3-ketoacyl-CoA synthase*, related to wax metabolism and embryo development.	1
	*CER*1 (*ECERIFERUM* 1), related to wax metabolism and embryo development	1
Embryo sac (7 unigenes)	*Unfertilized embryo sac* 1, 2	3
	*EDA*; *embryo sac development arrest* 7, 16, 39, 30	4
Ovule (1 unigenes)	*BEL1-like homeodomain transcription factor*, involved in regulation of ovule development in *Arabidopsis*	1
Endosperm (2 unigenes)	*ACR4 CRINKLY*4 (*Cr*4), belongs to cell fate-specifying genes and is required to specify aleurone cell.	1
	*VQ-motif containing protein*, regulates endosperm growth and seed size	1
Female gametophyte (2 unigenes)	*SLOW WALKER*1,2, related to female gametophyte , and essential for female gametogenesis	2
Pollen and male gameto- phyte (25 unigenes)	*Less adherent pollen 1* (*callose synthase* 5; *CALS*5)	1
	*NPG*1 (*no pollen germination* 1)	1
	*NPGR* (*no pollen germination related* )1, 2	2
	*AtPSKR*2; *Phytosulfokine receptor 2*, regulates pollen germination	1
	*DUO pollen 3-like protein*, is a key regulator of male germline development and embryogenesis	1
	*Villin headpiece*, is necessary for normal pollen tube growth.	2
	*VLN*1, 2 (*Vilin-like* 1, 2), related to pollen tube growth.	2
	*THE*1 (*THESEUS*1), inhibiting cell elongation during pollen tube/synergid cell recognition and in sensing cell wall integrity after damage.	1
	*extra sporogenous cells* (*EXS*), related to tapetum development	1
	*EMS*1,*EXCESS MICROSPOROCYTES*1	1
	*callose synthase* 1, 5, 7, 9, 10, 11, 12, putative	11
	*PAB*6;*POLY(A) BINDING PROTEIN* 6, related to male gametophyte	1
	*HUA enhancer* 2, related to the development of stamens and petals	1
Fertility and compatibility (3 unigenes)	*Male sterility MS5*	1
	*fringe-related protein*, related to fertility	1
	*arm repeat-containing protein*, related to incompatibility	1
Flower and flowering (16 unigenes)	*flowering locus* C *Variant*5, D	2
	*Early flowering* 3, 6	3
	Similar to *PIE*1; *photoperiod -independent early flowering*1	2
	*REF*6;*relative of early flowering* 6	2
	*Embryonic flowe*r 1, involved in the control of shoot architecture and flowering	1
	*CDPK-related protein kinase*, related to flowering time	1
	*FPA*; *Flowering time control protein*	1
	*PTL*; *petal loss*	1
	*PAN* (*PERIANTHIA*), controlling the number of floral organs such as calyx and petals	1
	*tesmin/TSO1-like CXC domain-containing protein*, required for organ formation in floral tissues.	2
Sexuality (1 unigenes)	GHMP kinase-related, primary determinant of sexual fate in *C. elegans*	1
Meiosis (7 unigenes)	*MLH*3 (*MUTL PROTEIN HOMOLOG* 3), related to meiosis.	2
	similar to *MRE*11 (*Meiotic recombination* 11)	1
	*Meiotic recombination protein DMC1 homolog*	1
	*MEI*1 (*meiosis defective* 1)	1
	*PMS*1 (*POSTMEIOTIC SEGREGATION* 1)	1
	*ZYP*1a, *Synaptonemal complex protein* 1, related to meiosis.	1
Root (15 unigenes)	*Root cap protein 2-like*	1
	*IRE* (*INCOMPLETE ROOT HAIR ELONGATION*)	3
	*Morphogenesis of root hair* 2	2
	*TRH*1 (*TINY ROOT HAIR* 1)	2
	*Root hair defective* 3	1
	Skewed roots; SKU5	1
	*ALF*4 (*ABERRANT LATERAL ROOT FORMATION* 4)	1
	*Roothairless*1, 3	2
	Similar to *Scarecrow,* essential for ground tissue organization in both root and shoot	1
	*ATIREG*2 (*IRON-REGULATED PROTEIN* 2), involved in iron-dependent nickel detoxification in roots	1
Nitrogen fixation (2 unigenes)	*vapyrin-like protein*, related to arbuscular mycorrhiza.	1
	*allantoate amidohydrolase,* related to nitrogen fixation.	1
Stem or meristem (11 unigenes)	*IMK*2 (*INFLORESCENCE MERISTEM RECEPTOR-LIKE KINASE* 2)	1
	*REPRODUCTIVE MERISTEM* 16	1
	*BAM*1 (*BARELY ANY MERISTEM* 1)	1
	*SPK*1 (*SPIKE*1), required for epidermal morphogenesis and the normal shape of cells and tissues.	1
	*Phytocalpain*, controls the proliferation and differentiation fates of cells in plant organ development	1
	similar to *SAB*, *suppressors of ABC*, related to epidermal hair and branch.	1
	*SGR*2 (*SHOOT GRAVITROPISM* 2)	1
	*Phototropic-responsive NPH3 family protein*, related to phototropic hypocotyl	3
	*NPH*3 (*NON-PHOTOTROPIC HYPOCOTYL* 3)	1
Leaf (5 unigenes)	*LNG*1 (*LONGIFOLIA*1)	1
	*SWP* (*STRUWWELPETER*), related to Cell numbers and leaf development. The levels of SWP, besides their role in pattern formation at the meristem, play an important role in defining the duration of cell proliferation.	2
	*EDR*1 (e*nhanced disease resistance* 1) a negative regulator of disease resistance and ethylene-induced senescence of leaves.	1
	*YLS*7, *leaf-senescence-related protein*	1

### GO functional annotation and classification of the *D. longan* transcriptome

To functionally categorize *D.longan* expressed genes, Gene Ontology (GO) terms were assigned to assembled unigenes. Based on BLASTx hits against the Nr database, Blast2GO [23] and WEGO [24] were used to obtain GO annotations and classifications according to molecular function, biological process, and cellular component ontologies.

**Figure 1:**
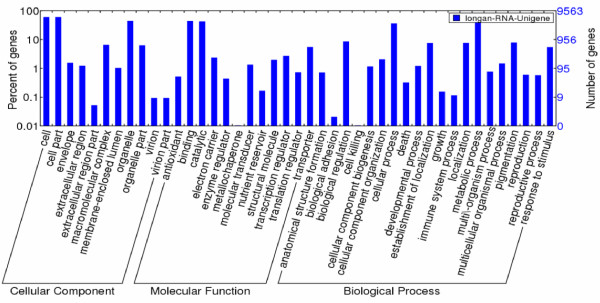
**Gene Ontology classification of the *D. longan *transcriptome.** Gene ontology (GO) term assignments to *D. longan* unigenes based on significant plant species hits against the NR database were summarized into three main GO categories (biological process, cellular component, molecular function) and 43 sub-categories.

Based on Nr annotations, 38,845 unigenes were assigned to the three main GO categories and 43 sub-categories, which included cellular process, metabolic process, death, development process, cell, organelle, antioxidant activity, catalytic activity, binding, enzyme regulator activity, transcription regulator activity, and translation regulator activity (Figure 1). Of the three main GO categories, cellular component was the most dominant category (17,417; 44.8%), followed by biological process (11,609; 29.89%) and molecular function (9,819; 25.28%) (Figure 1).

The biological process category was divided into 20 sub-categories. Among them, metabolic processes (3,786 unigenes; 32.6%) were the most highly represented, followed by cellular processes (3,438; 29.6%) and biological regulation (804; 6.9%). Only a few unigenes were assigned into sub-categories such as development process (114), death (31), growth (15), and immune system process (11) (Figure 1).

The cellular component category included 17,417 unigenes in 11 sub-categories, including cell (5,765 unigenes; 33.1%), organelle (4,259; 24.45%), macromolecular complex (632), envelope (149), extracellular region (119), and membrane-enclosed lumen (99) (Figure 1).

With respect to molecular function, 9,819 unigenes could be sub-categorized into 12 functional groups. These included 4,234 (43.12%) unigenes assigned to binding, followed by catalytic activity (4,073 unigenes; 41.48%) and transporter activity (528). In addition, a few unigenes were associated with transcription regulator activity (261), structural molecule activity (190), molecular transducer activity (128), translation regulator activity (70), antioxidant activity (50), enzyme regulator activity (42), nutrient reservoir activity (16), and metallochaperone activity (1) (Figure 1).

### COG functional annotation and classification of the *D. longan* transcriptome

**Figure 2:**
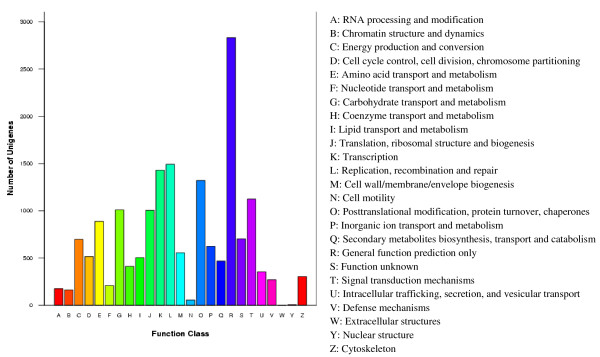
**COG function cof the D. longan transcriptome.** 17,118 unigenes showing significant homology to the COGs database at NCBI (E-value ≤ 1.0e-5) have a COG classification among the 25 categories.

The Clusters of Orthologous Groups (COG) database is based on a set of coding proteins with complete genomes and information about systematic evolutionary relationships of bacteria, algae, and eukaryotes. All longan unigenes were searched against the COG database to predict and classify by possible function. Overall, 17,118 (24.84%) unigenes were assigned to 25 COG categories (Figure 2).

Of the 25 COG categories, the cluster for General Function Prediction associated with basic physiological and metabolic functions represented the largest group (2,832; 16.54%), followed by Replication, recombination and repair (1,492; 8.72%), Transcription (1,428; 8.34%), Post-translational modification, protein turnover, chaperones (1,321; 7.72%), Signal transduction mechanisms (1,124; 6.57%), Carbohydrate transport and metabolism (1,009; 5.89%), Translation, ribosomal structure and biogenesis (1,005; 5.87%), and Amino acid transport and metabolism (887; 5.18%). A few unigenes were assigned to Cell motility (56; 0.33%), Nuclear structure (6; 0.04%), and Extracellular structures (1; 0.01%). In addition, 704 (4.11%) of longan unigenes were assigned into the Function Unknown cluster (Figure 2).

### KEGG functional classification of the *D. longan* transcriptome

The Kyoto Encyclopedia of Genes and Genomes (KEGG) database can be used to analyze gene products of metabolic processes and related cellular processes and to further research the genetics of biologically complex behaviors. To identify biological pathways in *D.longan*, unigenes were compared against the KEGG database using BLASTx; as a result, 17,306 unigenes were assigned to 199 KEGG pathways (Additional file 2).

**Figure 3:**
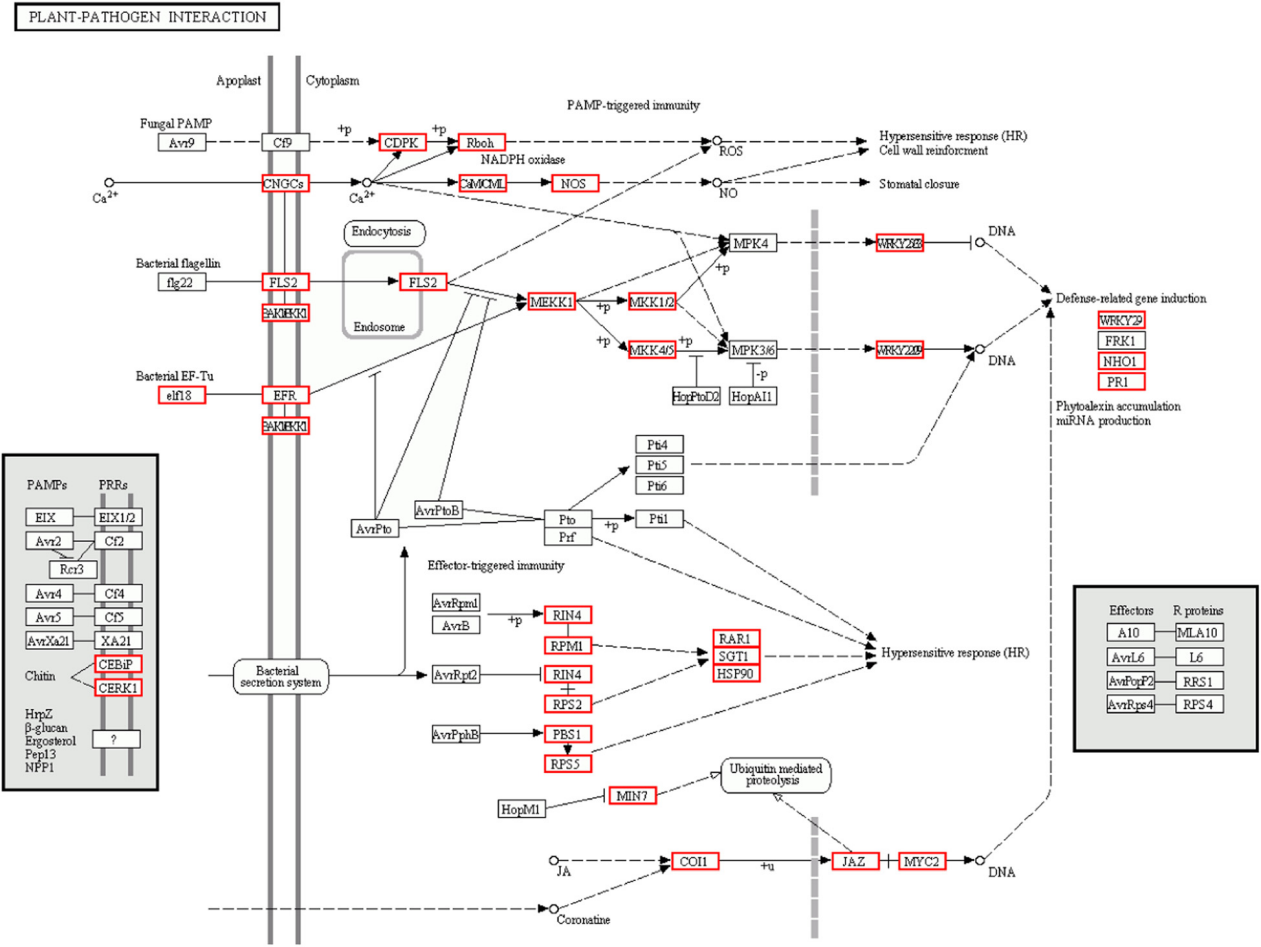
**Plant-pathogen interaction.** 1,410 (8.15%) unigenes were assigned to Plant-pathogen interaction pathways by KEGG. The longan unigenes involving in the pathways are in red boxs.

Among the 199 KEGG pathways, the pathways most represented by unigenes were metabolic pathways (3,942, 22.78%), primarily Starch and sucrose metabolism (492; 2.84%), Purine metabolism (406; 2.35%), Pyrimidine metabolism (334; 1.93%), Ubiquitin mediated proteolysis (427; 2.47%), Glycolysis/Gluconeogenesis (300; 1.73%), Cysteine and methionine metabolism (268; 1.55%), and Pyruvate metabolism (228;1.32%). In contrast, only a few unigenes were assigned to Thiamine metabolism (24; 0.14%), Riboflavin metabolism (26; 0.15%), Biotin metabolism (14; 0.08%), Vitamin B6 metabolism (14; 0.08%), C5-Branched dibasic acid metabolism (13; 0.08%), and Caffeine metabolism (13; 0.08%). In addition, 1410 (8.15%) of longan unigenes mapped to the Plant-pathogen interaction pathway (Figure 3), illustrating that many disease-resistance genes are expressed in longan EC.

**Figure 4:**
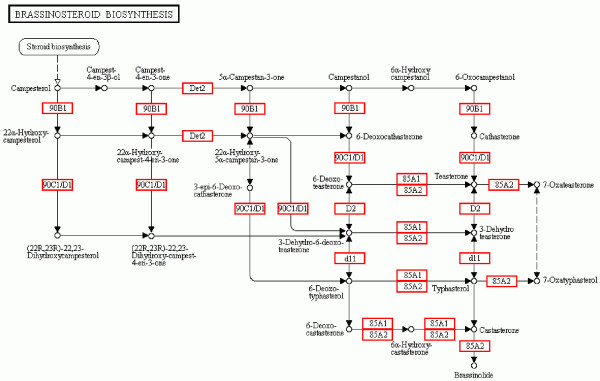
**Brassinosteroid biosynthesis.** 25 (0.14%) unigenes were assigned to brassinosteroid biosynthesis pathway by KEGG. The longan unigenes involving in the pathways are in red boxs.

Furthermore, 2,061 (11.91%) unigenes were classified into Biosynthesis of secondary metabolites pathways, including Stilbenoid, diarylheptanoid and gingerol biosynthesis (221; 1.28%), Flavonoid biosynthesis (188; 1.09%), Zeatin biosynthesis (155; 0.9%), Carotenoid biosynthesis (100; 0.58%), Biosynthesis of unsaturated fatty acids (94; 0.54%), Ubiquinone and other terpenoid-quinone biosynthesis (89; 0.51%), Terpenoid backbone biosynthesis (86; 0.5%), Steroid biosynthesis (78; 0.45%), Diterpenoid biosynthesis (56; 0.32%), Glucosinolate biosynthesis (54; 0.31%), Flavone and flavonol biosynthesis (52; 0.3%), Tropane, piperidine and pyridine alkaloid biosynthesis (48; 0.28%), Brassinosteroid biosynthesis (25; 0.14%), Folate biosynthesis (24; 0.14%) (Figure 4), and Anthocyanin biosynthesis (22; 0.13%).

**Figure 5:**
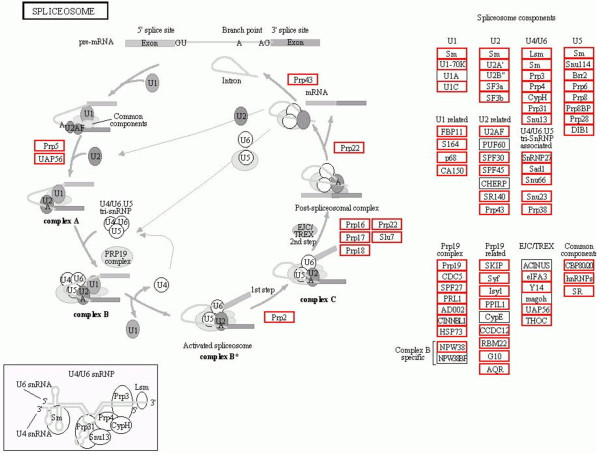
**Spliceosome.** 839(4.85%) unigenes were assigned to Spliceosome pathway by KEGG. The longan unigenes involving in the pathways are in red boxs.

In addition to the pathways mentioned above, many longan unigenes were associated with genetic information processing involving Spliceosome (839; 4.85%) (Figure 5), Ribosome (343; 1.98%), RNA degradation (268; 1.55%), Nucleotide excision repair (196; 1.13%), RNA polymerase (162; 0.94%), DNA replication (135; 0.78%), Base excision repair (126; 0.73%), Homologous recombination (106; 0.61%), Mismatch repair (104; 0.6%), Protein processing in endoplasmic reticulum (440; 2.54%), and Protein export (89; 0.51%).

**Figure 6:**
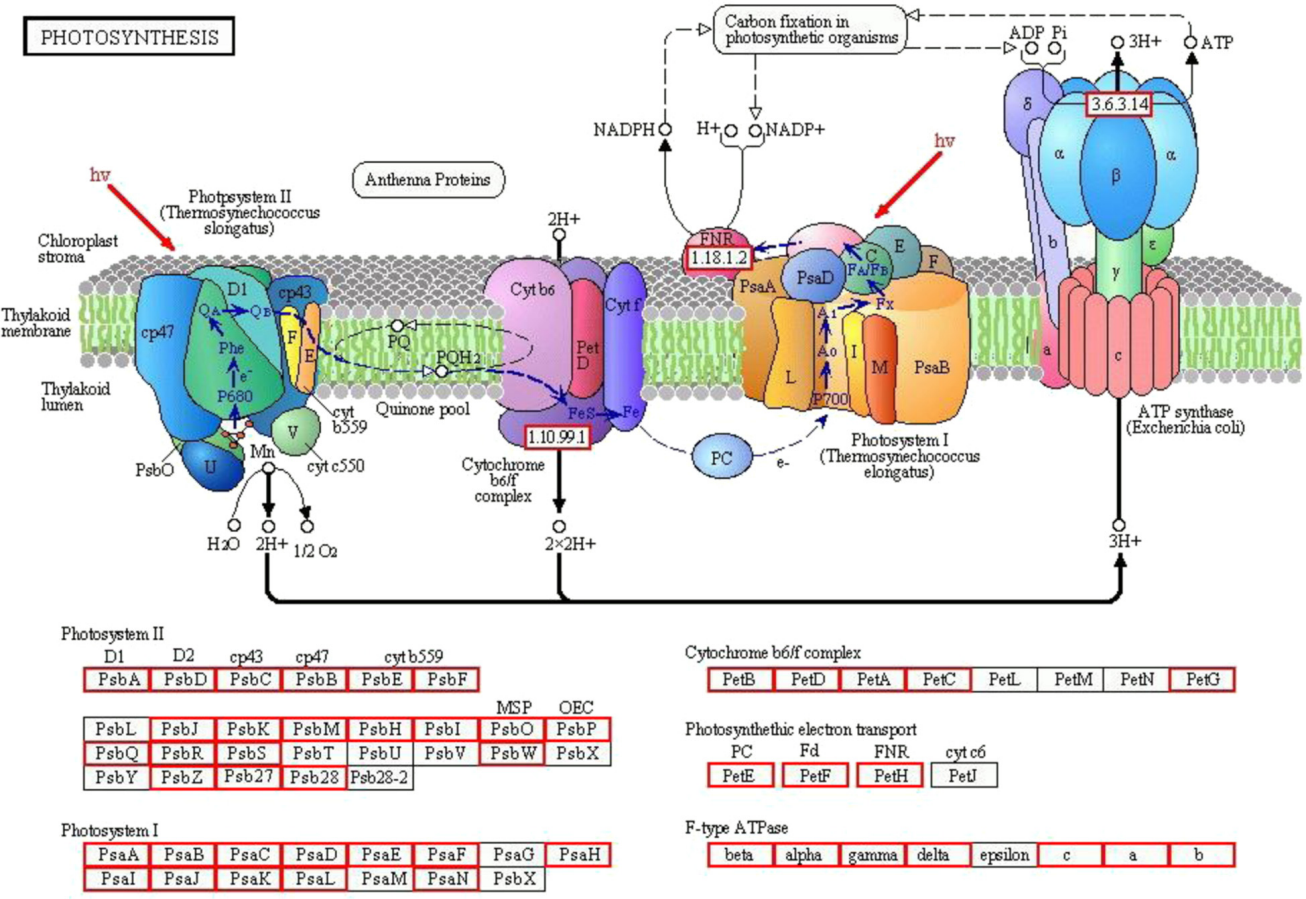
**Photosynthesis.** 85 (0.49%) unigenes were assigned to photosynthesis pathway by KEGG. The longan unigenes involving in the pathways are in red boxs.

Finally, longan unigenes were also involved in Carbon fixation in photosynthetic organisms (157; 0.91%), Photosynthesis (85; 0.49%) (Figure 6), and Photosynthesis- antenna proteins (15; 0.09%).

Taken together, the annotated longan unigenes provided valuable information for investigating specific processes, functions, and pathways involved in longan EC development, and allowed identification of novel genes in non-model organisms.

### Gene validation and expression analysis during longan SE using quantitative real-time PCR

**Figure 7:**
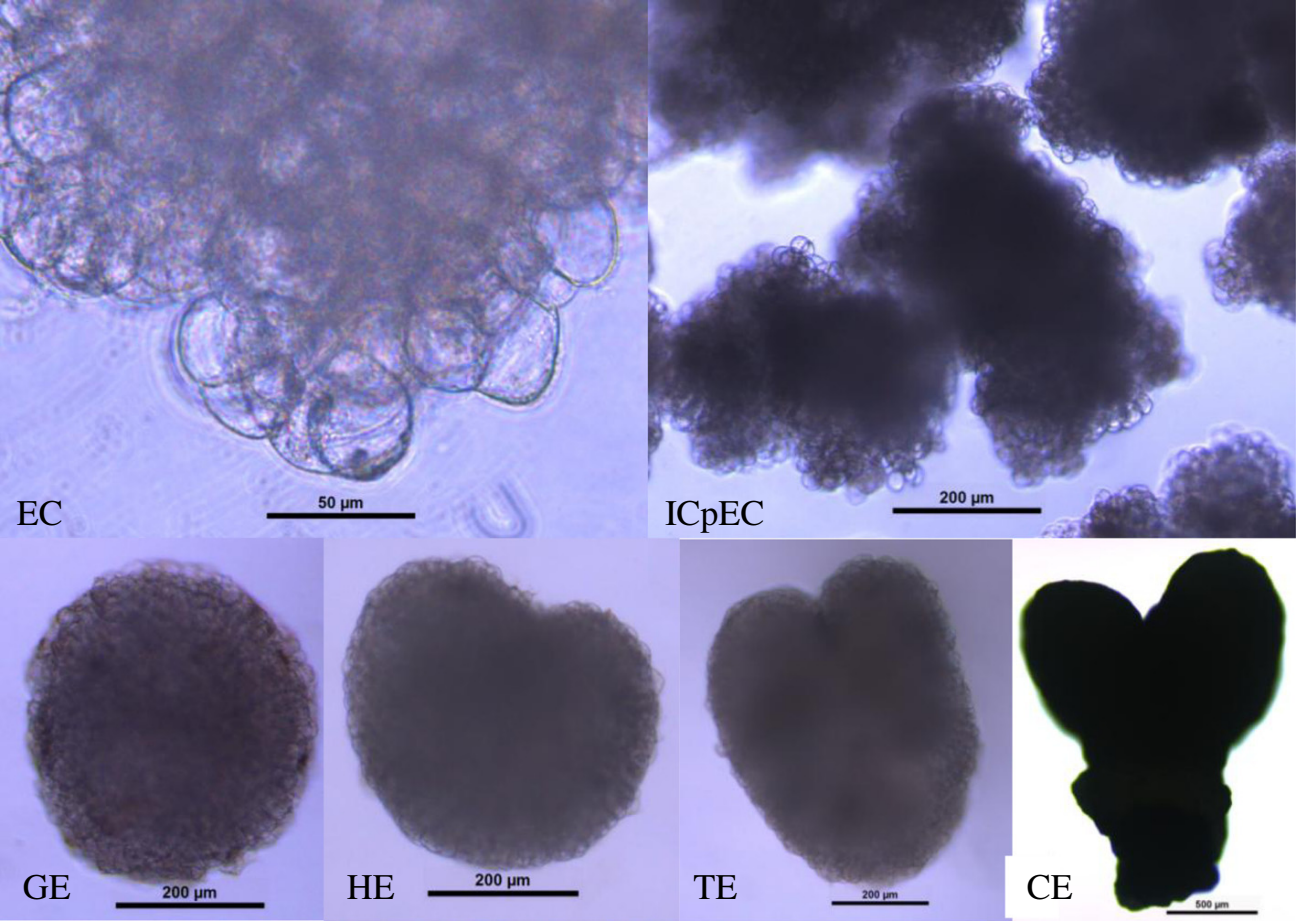
**Morphology of embryogenic calli and embryos during the six sequential developmental stages of longan SE.** The bars in each phenotypic class are indicated at the middle of each image. The morphology of embryogenic cultures friable-embryogenic callus(EC), incomplete compact pro-embryogenic cultures (ICpEC), globular embryos(GE), heart-shaped embryos(HE), torpedo-shaped embryos (TE), and cotyledonary embryos (CE) were observed using an inverted Leica DMIL LED microscope, except for EC(bar=50 μm) and CE(bar=500 μm), the bars of others are 200 μm; EC, ICpEC and GE, were cultured on MS medium supplemented with 1 mg/L, 0.5 mg/L, and 0.1 mg/L 2,4-D, respectively; and the HE, TE and CE were cultured on MS medium.

To experimentally confirm that unigenes obtained from sequencing and computational analysis were indeed expressed, 23 unigenes longer than 1000 bp, including 5 embryogenesis-related genes (*PPR1*_Unigene68247, *PPR2*_ Unigene 68600, *EMB1*_Unigene68678, *EMB2*_Unigene68326, and *EMB3*_Unigene 1123), 13 reproductive growth-related genes (*REF6*_Unigene14918, *GHMP1*_Unigene 10997, *GHMP2*_Unigene67027, *FRP*_Unigene68243, *EDA7*_ Unigene65846, *BEL1-like*_ Unigene68544, *SWA1*_Unigene 68185, *SWA2*_Unigene 68513, *NPG1*_Unigene68796, *NPGR1*_Unigene15267, *NPGR2*_Unigene 68058, *VLN1*_Unigene4052 and *VLN2*_ Unigene67205), and 5 vegetative growth-related genes (*MRH2*_Unigene 12452, *AAH*_Unigene68023, *SPK1*_Unigene68865, *SWP1*_Unigene68809 and *SWP2* _Unigene68236), were selected for qPCR analysis across the six sequential developmental stages of longan SE: friable-embryogenic callus (EC), incomplete compact pro-embryogenic cultures (ICpEC), globular embryos (GE), heart-shaped embryos (HE), torpedo-shaped embryos (TE), and cotyledonary embryos (CE) (Figure 7).

**Figure 8:**
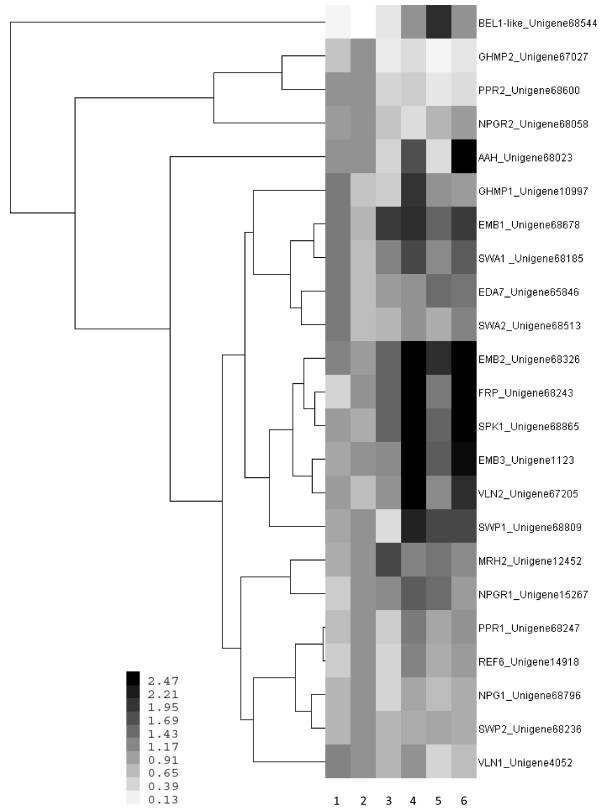
**Cluster analysis of expression profiles of 23 selected unigenes during longan SE.** The bar represents the scale of relative expression levels of unigenes, and colors indicate relative signal intensities of unigenes. Each column represents a sample, and each row represents a single unigene. Samples: 1. EC; 2. ICpEC; 3. GE; 4. HE; 5. TE; 6. CE.

Based on the analyzed qPCR data, all selected unigenes were expressed at varying levels in different embryogenic tissues (Figure 8). *MRH2*, *NPGR1*, *PPR1, REF6*, *NPG1*, *SWP2*, and *VLN1* were expressed throughout the different tissue culture developmental stages, although no significantly expression profiles were observed. Expression levels of *EMB2*, *EMB3*, *FRP*, *SPK1*, *VLN2*, *AAH*, and *SWP1* were low in TE, and high in HE and CE, while *BEL1-LIKE* exhibited the highest expression in TE. *GHMP2*, *PPR2*, and *NPGR2* were highly expressed in ICpEC, *GHMP1*, *EMB1*, and *SWA1* showed strong expression in HE, moderate expression in EC, and weak expression in ICpEC. *EDA7* and *SWA2* were highly expressed in EC. These results confirm that differential expressions of these unigenes have potential roles during longan SE.

## Discussion

### Feasibility of Illumina paired-end sequencing and assembly for non-model species with unsequenced genomes such as longan

Understanding the dynamics of plant transcriptomes is helpful for studying the complexity of transcriptional regulation and its impact on phenotype [25]. Transcriptome sequencing is one of the most important tools for gene discovery and expression pattern identification, but traditional EST sequencing based on the Sanger method is time-consuming and costly. Because of their high throughput, accuracy, and low cost, next-generation sequencing technologies, such as Illumina/Solexa, 454, and MPSS, have been used successfully for plant genomic and transcriptomic analyses in many organisms [26-28]. In this study, approximately 64 million clean reads (5.84 Gb of nucleotides) were obtained from longan EC using Illumina HiSeq 2000 sequencing and assembled into 68,925 unigenes, more than that reported for plants such as *S.indicum*[29], *T.chinensis*[30], *C.sinensis*[31], *G. hirsutum*[32] and *I.batatas*[33] using the same technology. Compared with previous studies, these sequences produced shorter unigenes (mean = 448 bp) than those assembled from *Sesamum* (629 bp), *Taxus* (1077 bp), *I.batatas* (581 bp), *Poncirus trifoliata* (1000 bp; from MPSS) and *Jatropha curcas* (916 bp; from 454) [34], but longer than those generated from *C.sinensis*[31], *Fagopyrum* (341 bp; 454) [35], and maize (218 bp; 454) [36]. More importantly, 5,696 (8.26%) of the assembled unigenes were longer than 1000 bp. These results demonstrate that Illumina sequencing technology can be an effective tool for gene discovery in non- model organisms. Moreover, 58.95% (40,634) of the putative protein unigenes showed significant similarity to known plant proteins in databases, a higher percentage than that reported for *S.indicum* (54.03%) [29], *I.batatas* (46.21%) [33], and *Epimedium sagittatum* (38.50%) [37]. On the other hand, average unigene length in our study was shorter than that obtained for most plant species, and there were many unassembled reads; difficulties with the *de novo* transcriptome assembly may be attributed to various factors, such as short sequence fragments, assembly options, genes expressed at low levels, repetitive sequences, alternative splicing, and lack of a reference genome [33].

### Quantity and variety of genes expressed in longan EC

Plant callus transcriptomes have been obtained for a number of species, including rice [11,12], *Populus*[13,14], *A.thaliana*[15,16], *G.hirsutum*[17], *S.tuberosum*[18] and *E.guineensis*[19]. In this study, 68,925 unigenes from longan EC were assembled, more than the number reported from calli of rice in one study (2,259 ESTs) [11], *G. hirsutum* (242 ESTs) [17] and *S.tuberosum* (14,744 unigenes) [18], but less than from rice in another study (218 million unigenes) [12], *Populus* (86,777 unigenes) [14], and *A.thaliana* (1,959,539 unigenes) [15]. The above studies failed to obtain global expression profiles of callus genes, either because there was insufficient transcriptome information or because no further analysis was conducted.

Studies have shown that plant SE is morphologically and molecularly similar to zygotic embryogenesis [4-7]. Just as a plant zygotic embryo, the most complex plant organ, epitomizes the entire plant, so too a plant somatic embryo can be considered to represent an entire plant. In our study, we uncovered 68,925 unigenes, the majority of which reflected expression of genes required for plant *in vitro* embryogenesis, such as Pentatricopeptide repeat proteins (PPR) and *Embryo defective* (EMB) family genes. A previous study has shown that mutations of different PPRs have distinct impacts on embryo morphogenesis [38]. In our study, 253 PPR unigenes longer than 1000 bp were identified, and two highly abundent PPR unigenes were further confirmed and found to be expressed throughout longan SE. *PPR1*_Unigene 68247 was highly expressed in ICpEC and HE, while *PPR2*_Unigene 68600 mRNA was abundant in EC and ICpEC. These results suggest the involvement of these genes during early developmental stages of longan SE. In *Arabidopsis*, 250 EMB genes have been confirmed to be required for normal embryo development [39], with *EMB175* displaying aberrant cell organization and undergoing morphological arrest before the globular-heart transition [38]. In our study, three *EMB* unigenes longer than 1000 bp—*EMB1*_Unigene68678, *EMB2*_Unigene68326, and *EMB3*_Unigene1123—were verified and strongly expressed in HE and CE. In addition, *EMB1*_Unigene68678 was also highly expressed in GE and moderately detectable in EC and TE, while *EMB2*_Unigene68326 and *EMB3*_Unigene1123 exhibited moderate expression in GE and TE. High expression levels of these selected EMBs in longan HE and CE indicate their possible roles in the development of longan SE.

Surprisingly, however, there were also many unigenes expressed in EC associated with reproductive growth characteristics (such as flowering, gametophytogenesis, and fertility), and vegetative growth (such as root and shoot growth). In our study, 13 reproductive growth-related genes longer than 1000 bp were confirmed by qPCR during the developmental stages of longan SE. For example, the fertility-related *FRP* (fringe-related protein), was strongly expressed in HE and CE, and also weakly in TE. Genes related to pollen growth—*NPGR* (no pollen germination), *NPGR* (no pollen germination-related), and *VLN* (Vilin-like)—displayed ubiquitous but weak expression during longan SE. *NPGR2*_Unigene68058 was highly expressed in ICpEC, and *VLN2*_Unigene67205 was accumulated in HE and CE, but barely detectable in TE. *SWA1/2*, essential for gametogenesis in *Arabidopsis*[40,41], were also differentially expressed during longan SE; while they were both highly expressed in EC, while *SWA1*_Unigene68185 was also expressed in HE. The GHMP kinase enzyme family, a primary determinant of sexual fate in *Caenorhabditis elegans*[42], was also detected in our study. *GHMP1*_Unigene10997 expression was high in HE, and *GHMP2*_Unigene67027 transcripts accumulated in ICpEC. *BEL1-LIKE*, is required for cytokinin and auxin signaling during ovule development in *Arabidopsis*[43], was expressed highly in TE and moderately in HE and CE, but was barely detectable in ICpEC; this suggests it may play a major role in longan SE during late embryonic stages. *EDA7*, related to embryo sac development, was strongly expressed in EC, TE, and CE. These results all demonstrate that these 13 reproductive growth-related genes also play roles during longan SE development. Five vegetative growth-related genes were also chosen for qPCR analysis across the six sequential developmental stages of longan SE. These genes included *MRH2* (morphogenesis of root hair 2), which is likely involved in polarized growth of root hairs in *Arabidopsis*[44], *REF6* (relative of early flowering 6), which plays divergent roles in the regulation of *Arabidopsis* flowering [45], and *SWP* (STRUWWE LPETER), which plays an important role in defining the duration of cell proliferation [46]. All of these genes were expressed at varied levels in different embryogenic tissues, suggesting their wide involvement in various developmental stages during longan SE. In particular, *SWP1*_Unigene68809 was highly expressed during late stages of longan SE, but was barely detectable in GE. In addition, *SPK1* (SPIKE1), required for normal cell shape control and tissue development [47], and *AAH* (allantoate amidohydrolase), related to nitrogen fixation, were both strongly expressed in HE and CE. The number and variety of expressed genes in EC suggests that EC practically reflects the entire SE profile, and even that of the entire plant. In addition, EC might be considered as a “gene pool” for isolating various plant target genes, such as genes related to flowering, pollen development, root and shoot growth, and plant-pathogen interactions. This EC dataset provides new candidates with possible roles in longan somatic embryogenesis.

In *Arabidopsis*, the largest number of unannotated signatures was found in callus: 1,655 (6.7%), compared with 884 in inflorescences, 935 in leaves, 1,089 in roots, and 907 in siliques [15]. In our study, we also found many unigenes (704; 4.11%) with unknown function, demonstrating how little is known about the biology of undifferentiated plant cells. Thus, the EC transcriptome can be used to more effectively discover new genes in longan.

Using Solexa sequencing, 27% of identified unigenes in *Populus euphratica* callus were found to be differentially expressed in response to salt stress; these genes were mainly involved in transport, transcription, cellular communication, and metabolism [14]. During *Arabidopsis* callus development, 241 genes were found to be up-regulated and 373 to be down-regulated. The most highly up-regulated genes encoded an unknown protein (At3g60420) and acireductone dioxygenase (At2g26400), and the most highly down-regulated genes included a DR4 protease inhibitor (At1g73330), two peroxidase genes (At5g17820 and At5g666390), two pEARLI 1 genes (At4g12480 and At4g12470), and two that encoded subtilases (At5g59090 and At5g44530) [16]. In rice callus cells, 16,000 expressed genes were identified using the microarray suite (MAS) 5.0 detection algorithm [48]. These studies demonstrate that a large number of genes involved in various biological and metabolic pathways are expressed in plant EC. In our study, 17,306 (25.11%) unigenes were assigned to 199 KEGG pathways, including Metabolic pathways, Plant-pathogen interactions, Biosynthesis of secondary metabolites, and Photosynthesis and Genetic information processing-related pathways. These results lay a foundation for further identification of longan EC-related genes.

## Conclusions

In summary, our study generated the first large-scale transcriptome dataset of longan EC. In addition, the types and quantities of genes expressed in longan, as well as their functions, classification, and metabolic pathways, were revealed for the first time. Twenty-three unigenes related to embryogenesis and reproductive and vegetative growth were differentially expressed in various embryogenic cultures, indicating their possible roles in longan SE. This transcriptome dataset provides new insights into molecular processes in *D. longan*.

## Methods

### Plant materials and RNA isolation

The synchronized cultures, consisting of friable-embryogenic callus (EC), incomplete compact pro-embryogenic cultures (ICpEC), globular embryos (GE), heart-shaped embryos (HE), torpedo-shaped embryos (TE), cotyledonary embryos (CE) of *D. longan* ‘Honghezi’, were generated as detailed in [8-10,49,50] and stored at −80°C for later use. Total RNAs were extracted from longan embryogenic cultures using Trizol Reagent (Invitrogen, USA). The resulting samples were treated with DNase I to remove any genomic DNA. Extracted RNAs were quantified using an Agilent 2100 bioanalyzer (Agilent Technologies) and checked for integrity using denaturing agarose gel electrophoresis with ethidium bromide staining. Only RNA samples with A260/A280 ratios between 1.9 and 2.1, RNA 28S:18S ratios higher than 1.0, and RNA integrity numbers (RINs) ≥ 8.5 were used in subsequent analyses.

### Longan EC cDNA library construction and Illumina sequencing

For Illumina sequencing, Poly(A)^+^ RNA was isolated from longan EC total RNA using Dynal oligo(dT)_25_ beads according to the manufacturer’s instructions. Following purification, fragmentation buffer was added to cleave the mRNA into short fragments. First-strand cDNA was synthesized using these short fragments as templates, along with SuperScript III reverse transcriptase and N6 random hexamer primer. Second-strand cDNA was then synthesized using buffer, dNTPs, RNaseH and DNA polymerase I. The resulting double-stranded cDNA was subjected to end-repair using T4 DNA polymerase, DNA polymerase I Klenow fragment, and T4 polynucleotide kinase, and ligated to adapters using T4 DNA ligase. Adaptor-ligated fragments (200 ± 25 bp long) were purified using a QiaQuick PCR extraction kit and eluted with EB buffer. After analysis using agarose gel electrophoresis, suitable fragments were selected as templates for PCR amplification. Sequencing of the resulting longan EC cDNA library was carried out with an Illumina HiSeq 2000 system.

### Data filtering and *de novo* assembly

Following sequencing of the EC cDNA library, deconvolution and quality value calculations were performed on the resulting raw images. Before assembly, high- quality clean reads were generated from the raw reads by removing adapter sequences, duplicated sequences, low-quality reads with ambiguous bases (‘N’), and reads with more than 10% of Q-values < 20 bases. All subsequent analyses were based on clean reads. Transcriptome *de novo* assembly was performed using SOAPdenovo v1.03 (http:// soap.genomics.org.cn). First, high-quality clean reads with a certain length of overlap were combined by SOAPdenovo into longer fragments with no unknown sequences (‘N’) between them. Contigs were then joined into scaffolds using paired-end information. Finally, paired-end reads were used again for gap filling of scaffolds to obtain sequences with the smallest number of Ns and which could not be extended on either end. The resulting sequences were defined as unigenes. The entire set of reads used for final assembly was submitted to the NCBI Sequence Read Archive under the accession n° SRA050205.

BLASTx alignment with a cut-off *E*-value of 1 × 10^-5^ was performed between unigenes and Nr, Swiss-Prot, KEGG, and COG databases, and all plant proteins in the databases were taken into consideration in the search for homology. The best results from the alignment were used to predict unigene coding regions and direction (i.e., 5’ to 3’). When results from different databases conflicted, a priority order of Nr > Swiss-Prot > KEGG > COG was followed. Unigenes without homologs in any of the above databases were scanned using ESTScan [51] to predict coding regions and sequence direction.

### Gene annotation, classification, and metabolic pathway analysis

To assign putative functions to longan unigenes, various bioinformatics approaches were used for further annotation, classification, and metabolic pathway analysis. First, the unigenes were aligned to Nr and Swiss-Prot protein databases using BLASTx (*E*-value < 1 × 10^-5^), retrieving protein functional annotations. To better understand the annotation and distribution of the longan gene functions, Blast2GO [23] was used in conjunction with the Nr annotations to retrieve GO annotations of longan unigenes. WEGO software [24] was then used for GO functional classification of all longan unigenes according to molecular function, biological process, and cellular component ontologies. To predict and classify possible functions, longan unigenes were also compared against the COG database. The biological interpretation of longan unigenes based on the KEGG database was further extended by assigning them to metabolic pathways using BLASTx.

### Gene validation and expression analysis by real-time quantitative PCR

Twenty-three unigenes with potential roles in longan SE were chosen for validation using real-time quantitative PCR (qPCR) with gene specific primers designed using DNAMAN 6.0. Relative mRNA levels from each unigene in RNA isolated as described above from six *D.longan* tissue samples (EC, IcpEC, GE,HE,TE and CE) were quantified with respect to internal standards [2]. All reactions were performed in triplicate in a LightCycler 480 qPCR instrument (Roche Applied Science, Switzerland), with a dissociation curve used to control for primer dimers in the reactions. Abundances of the 23 unigenes were calculated relative to the expression of reference genes *DlFSD*1a, *EF-1a*, and *eIF-*4a. Gene names, primer sequences, product sizes, PCR efficiencies, and annealing temperatures are given in Additional file 3.

## Abbreviations

EC: Embryogenic callus; SE: Somatic embryogenesis; DDRT-PCR: Differential display reverse transcription PCR; qPCR: Real-time quantitative PCR; EST: Expressed sequence tag; MPSS: Massively parallel signature sequencing; SSH: Suppression subtractive hybridization; CDS: Protein coding sequences; Nr: Non-redundant protein; GO: Gene ontology; KEGG: the Kyoto encyclopedia of genes and genomes; COG: the Clusters of Orthologous genes; EMB: *Embryo defective*; MEE: *Maternal effect embryo arrest.*

## Competing interests

Both authors declare that they have no competing financial interests.

## Authors’ contributions

LZX conceived the study, participated in its design and coordination, and helped to draft the manuscript. LYL participated in the study design, carried out the experimental work, and wrote the manuscript. Both authors read and approved the final version of this manuscript.

## Supplementary Material

Additional file 1 **Length destribution of contings, scaffolds and unigenes from *D. longan *embryogenic callus.** Click here for file

Additional file 2 17,306 longan unigenes assigning to 199 KEGG pathways. Click here for file

Additional file 3 The selected gene names, primer sequences, product sizes, PCR efficiencies, and annealing temperatures. Click here for file
